# Case report: Reconstruction with thyrohyoidopexy in a dog presented with complete laryngo-tracheal separation

**DOI:** 10.3389/fvets.2025.1482151

**Published:** 2025-02-12

**Authors:** José Diogo Dos-Santos, Luís Belo, Mariana Monteiro, Lisa A. Mestrinho

**Affiliations:** ^1^I-MVET, Research in Veterinary Medicine, Faculty of Veterinary Medicine, Lusófona University-Lisbon, University Center, Lisbon, Portugal; ^2^Veterinary and Animal Research Center (CECAV), Lusófona University, Lisbon University Center, Lisbon, Portugal; ^3^Associate Laboratory for Animal and Veterinary Sciences (AL4AnimalS), Lisbon, Portugal; ^4^VetOeiras – Veterinary Hospital IVC Evidensia, Oeiras, Portugal; ^5^CIISA – Centre for Interdisciplinary Research in Animal Health, Faculty of Veterinary Medicine, University of Lisbon, Lisbon, Portugal

**Keywords:** laryngeal trauma, laryngeal separation, oropharyngeal anatomy, thyrohyoidopexy, virtual surgical planning

## Abstract

A 2-year-old Norfolk Terrier purebred female dog was presented for urgent attention after strangulation secondary to elevator entrapment. The traumatic event caused a complex laryngopharyngeal lesion with total laryngotracheal and esophageal separation from the hyoid bone and pharynx. Reconstruction was performed from the posterior pharyngeal wall, and all layers, mucosa, muscles and ligaments were repaired. A thyrohyoidopexy was done using nonabsorbable sutures to reinforce the thyrohyoid membrane reconstruction and prevent reseparation in the immediate postoperative period. The patient's fully recovery was gradual but uneventful, with occasional cough resolving within 2 months.

## Case description

A 2-year-old, 6.5 kg, intact female Norfolk Terrier presented to a referral hospital with inspiratory dyspnea, tachypnea and hemoptysis. The animal was still ambulatory. The owner reported acute trauma due to strangulation. The dog's leash stuck in the elevator while descending, causing entrapment of the dog's neck against the floor.

On the initial clinical examination, the animal was lethargic but conscious, with pale mucous membranes, delayed capillary refill time and severe inspiratory stridor. The skin was intact, but cervical emphysema was palpable and especially visible upon expiration.

Emergency treatment was initiated with flow-by-oxygen supplementation. This was followed by fluid therapy with lactated Ringer's solution and the intravenous (IV) administration of methadone (0.3 mg/kg) and methylprednisolone (1 mg/kg). A complete blood count, serum biochemistry profile, ionogram, and lactate levels did not reveal any abnormalities. The dog was then sedated with dexmedetomidine (1 μg/kg) and propofol (2 mg/kg) to establish a stable airway. Blood was identified in the oral cavity. The oropharyngeal anatomy was abnormal, the rima glottis was displaced caudally from the epiglottis, and a large hematoma, occupying the dorsal pharynx, was observed. Using a straight blade laryngoscope, a 4 mm diameter cuffed tube was introduced. After the establishment of a stable airway, the hemoglobin oxygen saturation, expired carbon dioxide fraction, and arterial pressures were monitored within normal limits. Lateral cervicothoracic radiography revealed cranial and caudal displacement of the larynx and subcutaneous air around and ventral to the neck, which was consistent with emphysema near the larynx. A computed tomography (CT) study was performed using a Siemens Somatom go.Now (CN), with a 0.5 mm slice thickness, bone and soft tissue windows with pre- and post contrast enhancement. This revealed caudal displacement of the larynx (arrowhead) relative to the hyoid apparatus (arrow), the latter with an abnormal rostroventral orientation, with a large gap of 4.6 cm between the thyrohyoid bone and the thyroid cartilages (normally articulating with each other). The sternohyoid and thyrohyoid muscles were caudally retracted from the basihyoid bone, all of which caused dilatation of the oropharynx with ventral hypoattenuating material and fluid/gas dissecting bilaterally in the retropharyngeal region.

**Figure 1:**
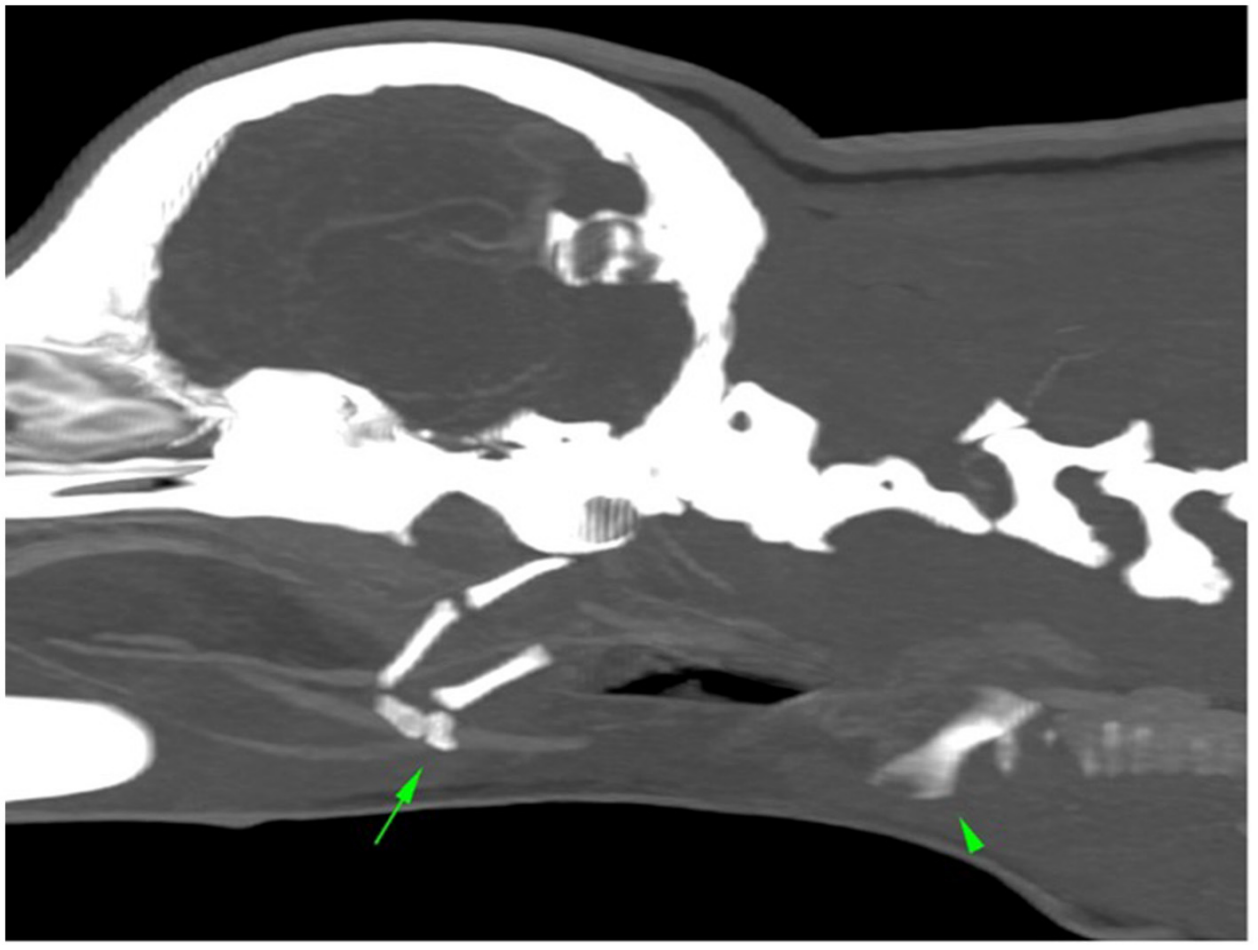
Computed tomography image: caudal displacement of the larynx (arrowhead) relative to the hyoid aparatus (arrow).

These findings were compatible with traumatic caudal laryngeal luxation, avulsion of the sternohyoid and thyrohyoid muscles and associated hemorrhagic/inflammatory changes in the oropharynx/nasopharynx and larynx (Figure 1).

During preoperative planning, a continuous infusion of dexmedetomidine (1 μg/kg/h) and fentanyl (4 μg/kg/h) was prepared for additional pain management and a minimal concentration of isoflurane was used to maintain hypnosis. Additionally, invasive arterial pressure monitoring was established after right metatarsal artery catheterization. Surgical preparation included ventral skin trichotomy and skin antiseptic preparation since the skin was intact. During the procedure, invasive mechanical ventilation was performed, controlled by volume, due to the presence of hypercapnia. The anesthetic plan was maintaining ventromedial rotation of the eyeball, absence of the palpebral reflex, and absence of jaw tone during all procedure. The anesthetic procedure proceeded without major hemodynamic or analgesic fluctuations. Mild hypothermia (37°C) was the main complication noted.

**Figure 2:**
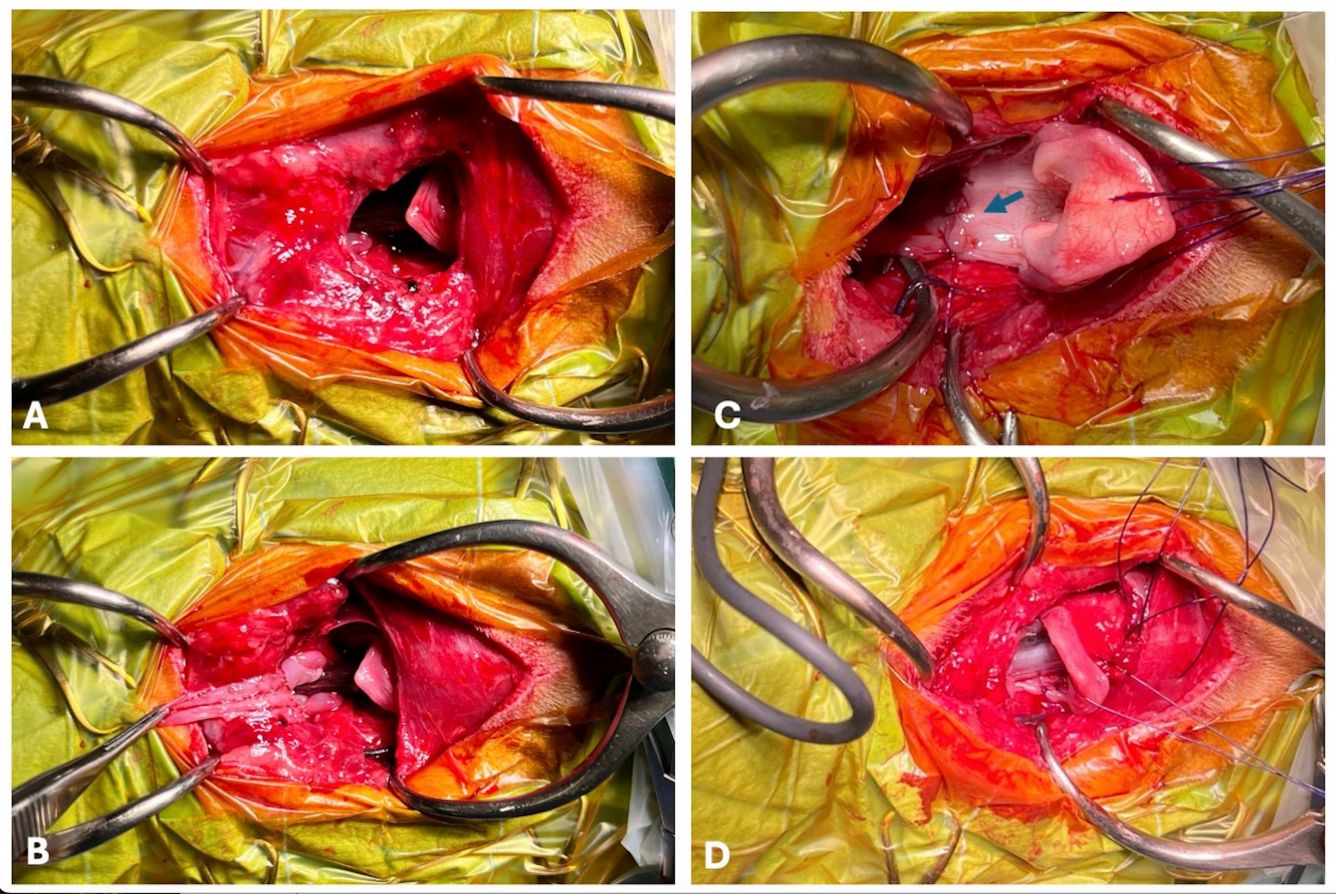
Intraoperative images immediately after the cutaneous incision **(A)**. The posterior pharyngeal wall was completely ruptured **(A, B)**. Moment of traction of the oropharyngeal mucosa, exposing the oropharyngeal isthmus and the posterior wall rupture and before removal of the orotracheal tube **(B)**. Posterior wall reconstruction (blue arrow) after removal of the orotracheal tube **(C)**. Orotracheal tube replacement and application of mattress sutures in the thyroid cartilage **(D)**.

The surgical procedure included a vertical median skin incision extending from the mandibular angular process line to mid cervical. After cutaneous access it was possible to inspect the cervical lesion, confirming the avulsed larynx and esophagus from the pharynx. Separation from the hyoid bone and pharynx was complete, which resulted from rupture of the thyrohyoid, sternohyoid and omohyoid muscles; the thyrohyoid membrane; and the median thyrohyoid ligament. Additionally, the posterior support of the larynx was also ruptured. This included the lateral thyrohyoid ligaments (both associated with thyrohyoid fracture at the level of the thyroid articulation) and the posterior pharyngeal wall. The absent insertions of the identified muscles from the hyoid bone pulled the larynx caudally, displacing both larynx and proximal esophagus ~4.6 cm as previously described. Displacement of the esophagus was identified only after surgical inspection since this finding was not accurately observed on CT (Figure 2).

**Figure 3:**
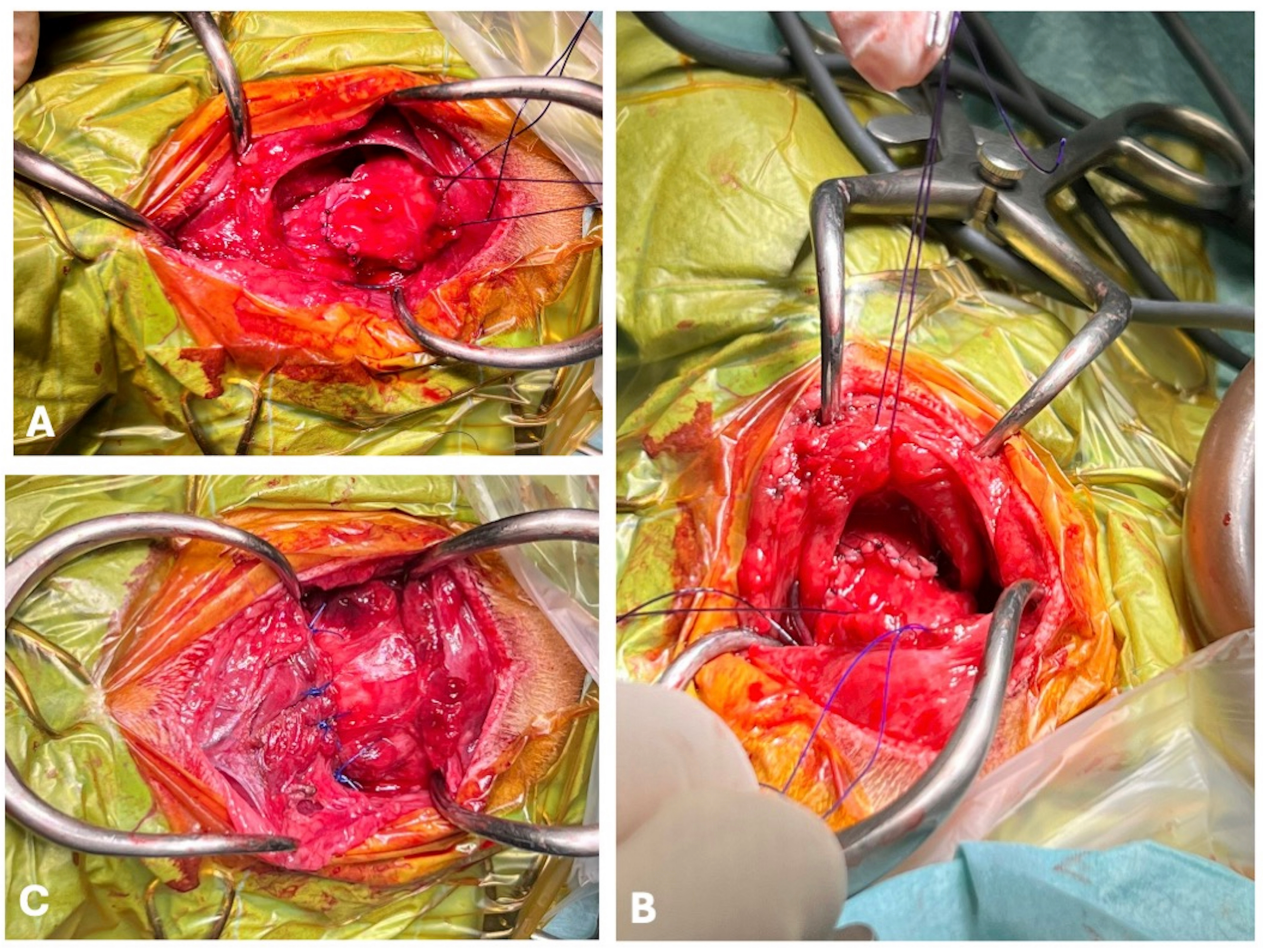
Reconstruction of the anterior pharyngeal wall **(A)** and thyrohyoidpexy using simple interrupted, nonabsorbable, sutures between the thyroid cartilage and the hyoid bone **(B, C)**.

Reconstruction was performed from the posterior wall (Figure 2C) by applying a simple interrupted pattern with a polyglyconate monofilament resorbable material. First, the continuity of the hypopharyngeal and esophageal mucosa was reestablished, avoiding knot exposure to the lumen. After the posterior wall reconstruction, the orotracheal tube was replaced with a larger tube (5.5). The new tube was maintained during the second part of the reconstruction. The ruptured thyrohyoid membrane, ligaments and muscles were sutured via the same resorbable monofilament suture in a simple interrupted suture pattern (Figure 3). All the sutures were placed before knot tying, which was performed from posterior to anterior. Before completion of the anterior pharyngeal suture, thyrohyoidopexy was performed via simple interrupted, polypropylene non-absorbable sutures with a 2/0 taper cut needle, and the thyroid cartilage was fastened to the hyoid bone at 4 points. Finally, for muscle reconstruction X-pattern or horizontal mattress sutures were applied. The esophagostomy tube was placed through a separate skin incision but was guided intraorally and carefully to avoid disrupting the reconstruction made. Finally, a double-lumen tracheostomy tube was placed to assure intraoperative anesthesia maintenance and postoperative airway patency.

After surgery cervical radiographs were taken to confirm the position of the feeding tube in the esophagus. The dog was taken to the hospitalization room for recovery and close monitoring. Oxygen supplementation was not necessary.

The tracheostomy tube inner canula was aseptically cleaned every 4–6 h after initial placement.

The patient was maintained under sedation during the postoperative period for 24 h, using a constant rate infusion (CRI) of fentanyl (2 μg/kg/h) and dexmedetomine (0.5–1 μg/kg/h), which was gradually reduced after 12 h. Other medications included paracetamol (10 mg/kg every 8 h), methylprednisolone (1 mg/kg every 24 h) and amoxicillin/clavulanic acid (12.5 mg/kg).

The tracheostomy tube was removed at 48 h after a brief sedation with propofol to assess laryngeal function and positioning. Adequate positioning and bilateral abduction were almost normal. Her breathing was normal, without laryngeal stridor. The stoma wound was left to heal by second intention. Water was offered first on the third day under close monitoring, followed by soft food. No dysphagia was noticed, and on the fourth day, the feeding tube was removed. A regime of six small meals of soft food per day was provided until discharge on the sixth day. The discharge prescriptions included robenacoxib (2 mg/kg every 24 h) for 5 days, amoxicillin/clavulanic acid (12.5 mg/kg every 12 h) for 5 days and paracetamol (10 mg/kg every 8 h) for 3 days. Further recommendations included three meals a day of soft food and cleaning the tracheal wound daily. At the first reevaluation, the owners reported occasional coughing, especially in the morning or when the patient was more excited.

One month after surgery, the animal was sedated to assess laryngeal function and position via endoscopy and CT. Normal bilateral arytenoid abduction was confirmed during inspiration, and a very small and smooth scar was observed inside the pharynx. The tracheostomy stoma was almost completely closed and the owner reported normal swallowing and normal breathing without any signs of dysphagia. However, cough was still noticed, albeit less frequently.

**Figure 4:**
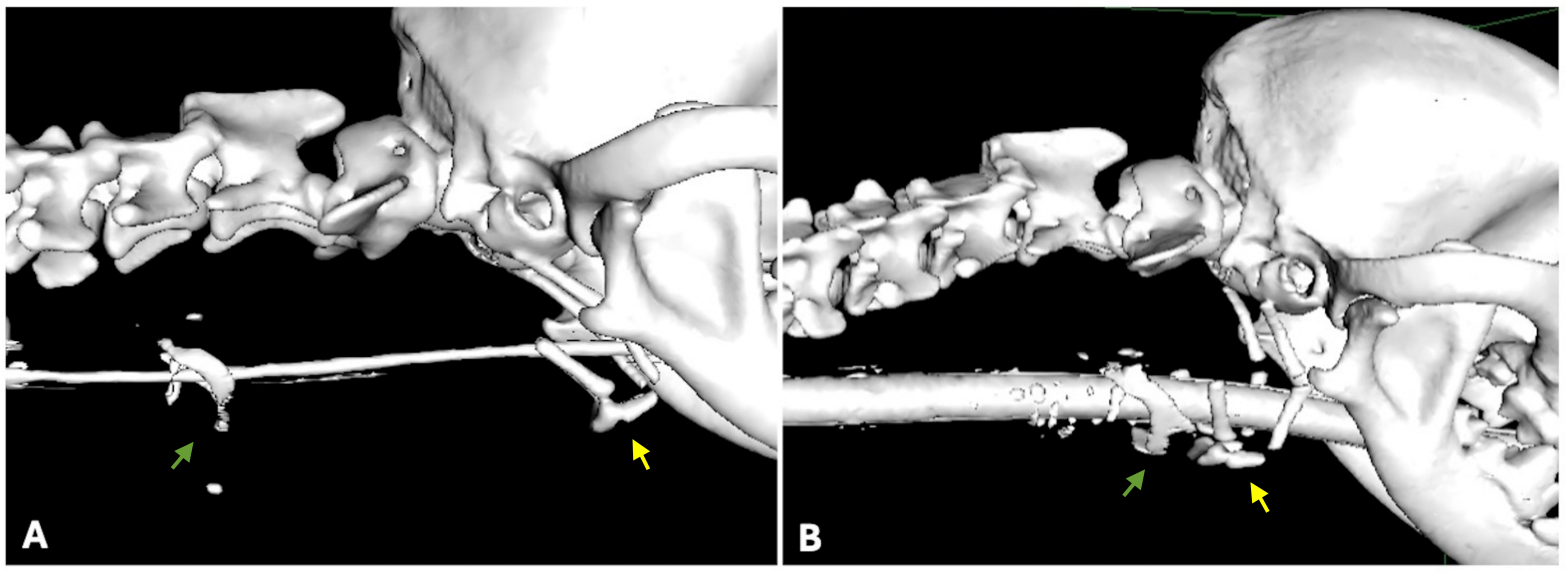
Segmentation obtained from DICOM images obtained via computed tomography. Spatial relationships between the thyroid cartilage (green arrow) and the hyoid bone (yellow arrow), before **(A)** and after surgery **(B)**.

CT revealed that the larynx and hyoid apparatus are normally positioned in the neck and are related to each other. The pre- and postoperative relationships between the thyroid cartilage and hyoid bone are illustrated in Figure 4. The sternohyoid and thyrohyoid muscles show an irregular contour, with heterogeneous and marked contrast enhancement at the level of the thyroid cartilage (green arrows in the central and right figures). At the level of C3-C4, at the ventral midline, there is a defect in the subcutaneous tissue, with focal dermal contrast enhancement, which is consistent with the tracheostomy site. Both medial retropharyngeal lymph nodes were mildly enlarged. No other anomalies were noted.

The dog is eating normally dry food and is still under surveillance. However, during the 9-months postoperative period, no further events have been registered thus far.

## Discussion

After a systematic search through the databases this is the first case report of complete separation of the larynx from the hyoid bone in the dog. Laryngeal trauma has been previously described in a case report and short case series, but authors did not find any similar lesions reported in the literature or with such a high severity, as presented here (1, 2). Laryngeal trauma and hyoid bone fracture have been previously described as secondary to bite injuries (2, 3). Most cases described in the literature included (more or less) severe laryngeal and hyoid fractures. In one cat from one case series, a complete separation of epiglottic and thyroid cartilages was mentioned (4).

This case report is highly relevant from the authors' perspective, given the rare and emergent nature of the case. The emergent nature of the lesion limits the examination workup and demands immediate and effective intervention.

The dog was presented with a severely compromised airway due to trauma, with signs of shock, hypoperfusion and cervical emphysema due to possible laryngo-tracheal rupture. This situation could rapidly lead to decreased cerebral oxygen delivery via compression of the cervical blood vessels or superior airway obstruction. Death can rapidly occur if the compressing forces are not removed or if the airway is not reestablished.

The immediate action was orotracheal intubation under mild sedation. This approach was performed with careful manipulation, given the fatal consequences of not establishing an effective airway. The possibility of performing a tracheostomy and cricostomy was not considered for two reasons: inaccessible cricothyroid kits and the need to position the dog in dorsal recumbency and neck extension. Given the suspicion of laryngotracheal and/or cricoid injury, endotracheal intubation in the sternal position was the maneuver that could lead to less impact on previous tissue lesions.

With respect to sedation, anesthesia and analgesia management, selected substances are in agreement with literature recommendations (5–7). The sedative options described include acepromazine or benzodiazepines. The first, when administered at low dosages, induces a reduction in the respiratory rate in dogs, accompanied by mild sedation and a prolonged duration of action. It is frequently employed to alleviate anxiety in patients and is particularly suitable for those with airway disease or dysfunction. However, its long-term plasma concentrations are challenging to predict, and it lacks analgesic effects. Benzodiazepines used alone seldom provide sufficient sedation in distressed patients (5). Opioids elicit dose dependent centrally mediated respiratory depression, which can be exacerbated by concurrent administration of other respiratory-depressant drugs (8). However, the degree of opioid-induced respiratory depression is generally minimal when appropriate doses are administered and can be easily managed, especially in patients undergoing anesthesia, and this patient was maintained with 100% oxygen. Since opioids acting on mu and kappa receptors offer profound analgesia and sedation in dogs, they are the preferred choice for surgical pain management, especially in the continuous infusion of fentanyl, used in this case. Its administration rate is tailored to the animal's needs, which is crucial in cases requiring pre, intra, and postoperative pain relief (9).

Furthermore, continuous infusion of dexmedetomidine, given its analgesic and sedative properties, can be advantageous for such patients, contributing to cardiovascular stability as well (7, 10).

In the present case, the dog's leash functioned as a separating structure moving the larynx from the hyoid apparatus apart from each other, as it was pulled against the neck. The excessive compressive force led to a ligament tear, with the thyrohyoid membrane, which connects the thyroid cartilage to the hyoid bone, being particularly vulnerable, along with the thyrohyoid muscle and ligaments.

CT is the recommended examination to assess the neck lesions properly (2) and was performed in this case. 3D reconstruction of the traumatized area via segmentation enhances visualization and improves the spatial anatomical understanding of the structures, which helps to clearly define the surgical plan. Segmentation is a process of dividing an image into specific anatomic structures, allowing separation of the hyoid and larynx from the remaining tissues. CT and the segmentation itself did not allow the identification of an esophageal separation, although this was suspected by the authors and later confirmed during the surgical exploration.

Anatomic reconstruction was a logical procedure, since it establishes the continuity of the airway, repositions and reconstructs muscles, ligaments and membrane tears, leading to the recovery of function. Additionally, this approach can correct subcutaneous emphysema and prevent possible pseudo mediastinum (11).

An esophageal tear occurred at the end of the hypopharynx. Anastomosis was performed routinely with simple interrupted sutures, avoiding leaving the knot toward the lumen. The knot exposed to the mucosa can potentiate early colonization and leakage. To avoid early aspiration, an esophageal tube was placed. The application of a gastric tube would be a reasonable option but was not considered in our case, because of there was no access to endoscopic gastrostomy placement and because it was possible to apply the esophagostomy tube away from the anastomotic site. The complication rate of esophageal tubes can reach 40% in dogs but severe complications are rare (12). No complications related to the esophageal tube were observed in this case.

Thyrohyoidopexy for laryngeal reconstruction is described in this case report. In the case of complete separation of the larynx from the hyoid bone, all the torn muscles, membranes and ligaments needed to be sutured. However, since most of the anchoring points of these structures are compromised, stability and reinforcement need to be added to the reconstructed sutured structures, especially to reduce tension on the mucosal suture. This procedure also addresses the concern that the compression from strangulation could lead to additional tissue ischemia, which could have been missed during reconstruction. Ischemia can lead to delayed dehiscence. This approach has been previously described in human patients with complete laryngeal separation due to strangulation, adapted according to the case (13, 14).

Temporary tracheostomy was necessary to allow the postoperatory recovery, bypassing postoperative edema, which would naturally compromise inspiration. In this case, temporary tracheostomy was unnecessary at the end of 48 h, although minor stridor was still audible.

Postoperative cough could be a reflex of incomplete recovery of the swallowing function or scarce laryngeal sensibility but not irreversible damage, since complete recovery after 2 months was observed. The complete recovery without dysphagia or chronic cough is suggestive that the strangulation did not irreversibly damage the nerves. Major vessels and nerves are the last structures to be damaged in a strangulation because of their intrinsic properties of elasticity and capacity to tolerate some level of stretching (15, 16).

In conclusion, this report describes the clinical presentation, diagnosis and management of a rare case of complete laryngeal separation after a strangulation incident. Surgical management after detailed 3D planning with reconstruction, including thyrohyoidopexy and esophageal and tracheal tube placement, assures complete recovery.

## Data Availability

The original contributions presented in the study are included in the article, further inquiries can be directed to the corresponding author.
